# AI-Enabled Digital Health Tool Implementation in Clinical Practice Settings: Umbrella Review

**DOI:** 10.2196/101509

**Published:** 2026-11-04

**Authors:** Amanda Gilbert, Angeline Gacad, Loni Parrish, Oliver Nguyen, Kea Turner, Willi L Tarver, Pallavi Jonnalagadda, Dennis Tsagli, Bowen Wu, Chenyang Lu, Rachel E Sachs, Oghosa Evbuomwan, Maura K Kepper

**Affiliations:** 1 Prevention Research Center, Burskey School of Public Health, Washington University in St. Louis, 1 Brookings Drive, St. Louis, MO, 63130, United States, 1 678 458 9379; 2 Department of Population Health, University of Kansas, Kansas City, KS, United States; 3 Department of Industrial and Systems Engineering, University of Wisconsin–Madison, Madison, WI, United States; 4 School of Nursing, University of North Carolina at Chapel Hill, Chapel Hill, NC, United States; 5 Department of Internal Medicine, College of Medicine, The Ohio State University, Columbus, OH, United States; 6 Department of Biomedical Informatics, College of Medicine, The Ohio State University, Columbus, OH, United States; 7 Department of Computer Science and Engineering, McKelvey School of Engineering, Washington University in St. Louis, St. Louis, MO, United States; 8 AI for Health Institute, Washington University in St. Louis, St. Louis, MO, United States; 9 School of Law, Washington University in St. Louis, St. Louis, MO, United States

**Keywords:** AI, implementation science, health care, digital health, determinants

## Abstract

**Background:**

AI has the potential to improve clinical decision-making, efficiency, and patient outcomes, yet adoption in routine clinical practice has been slower than anticipated. While prior studies have identified factors that influence AI implementation, a comprehensive synthesis grounded in implementation science frameworks is lacking.

**Objective:**

This study aimed to qualitatively and comprehensively describe barriers and facilitators to AI implementation in clinical care settings using an implementation science determinants framework, the Consolidated Framework for Implementation Research (CFIR), and to identify AI-specific determinants not fully captured by CFIR.

**Methods:**

We conducted an umbrella review of reviews examining determinants of AI implementation in clinical practice. In accordance with the PRIOR (Preferred Reporting Items for Overviews of Reviews) reporting guidance, we systematically searched PubMed, Scopus, and CINAHL for English-language reviews published between January 2013 and July 2025. Reviews were included if they addressed barriers to and facilitators of implementing AI-enabled digital health tools in clinical care settings. Determinants were deductively mapped to the updated CFIR and inductively analyzed to identify emergent AI-specific constructs. Review quality was assessed using the Joanna Briggs Institute critical appraisal checklist, and overlap among primary studies was evaluated using the GROOVE (Graphical Representation of Overlap for Overviews) tool.

**Results:**

Twenty-two reviews published between 2021 and 2025 were included, encompassing studies conducted from 1990 to 2025. Reviews varied in design, including systematic, scoping, rapid, narrative, and literature reviews. Overall review quality was mixed, with 9 (41%) reviews rated at 100% quality and 4 (18%) at 50% or lower. Primary study overlap was slight, with a corrected covered area of 0.4%. Determinants of AI implementation were identified across all CFIR domains. Prominent barriers at the innovation level included limited evidence of clinical impact, poor interpretability, complexity, lack of adaptability, and insufficiently representative data. Facilitators included perceived relative advantage, co-design with clinicians, and evidence of local clinical benefit. At the implementer level, low AI literacy, limited confidence, time constraints, and concerns about professional displacement were common barriers, while training and technical support facilitated adoption. Inner setting barriers included inadequate infrastructure, limited resources, poor workflow compatibility, and weak governance structures. Outer setting barriers included unclear policies, laws, and liability frameworks, whereas partnerships and cross-sector collaboration were frequent facilitators. Inductively identified AI-specific determinants included transparency, trust, accountability, model bias, data governance, model maintenance, robustness, generalizability, existential threat, and concern about loss of the human element in care.

**Conclusions:**

AI implementation in clinical settings is shaped by complex, multilevel, and AI-specific determinants that extend beyond traditional digital health considerations. Implementation science frameworks can be adapted to incorporate these constructs. Tailored, theory-informed strategies addressing technical, organizational, psychological, and regulatory factors are essential to support safe, equitable, and sustainable integration of AI into clinical care.

## Introduction

The integration of AI into clinical practice has the potential for profound transformation in modern health care that promises advancements that enhance both clinical outcomes and operational efficiencies [1]. AI systems, encompassing technologies such as machine learning (ML), deep learning, and natural language processing, are designed to replicate complex cognitive functions, enabling accurate information analysis and improved decision-making [2,3]. The deployment of AI may address pervasive systemic challenges within health care, including high workloads that lead to medical errors, staff shortages, and the unequal distribution of medical resources [2]. AI offers the potential for earlier disease detection, improved patient safety, better estimation of resource capacity, and the facilitation of personalized treatment plans [4]. For instance, AI-powered clinical decision support systems provide real-time, data-driven insights that assist clinicians in disease detection, diagnosis, and treatment, making AI use a pivotal strategy for enhancing the quality of patient care globally [1,3,5].

Despite the demonstrated capabilities and enthusiastic investment in AI technologies, integration into clinical practice has been slow and inconsistent, limiting its effect on transforming health care outcomes [1,4]. Barriers to AI implementation, including psychological and technical barriers, are being identified. These include the lack of transparency or the “black box” nature of AI and concerns among health care professionals regarding job displacement, erosion of professional identity, and overreliance (manifesting as skepticism, reluctance, anxiety, and fear) [3,6]. Other barriers relate to environmental determinants, such as health care infrastructure and data availability, and the legal and ethical frameworks necessary for AI use and evaluation, which complicate implementation and sustainment across complex health systems [1,7]. However, the potential opportunities for reducing workloads, improving clinical care outcomes, and increasing efficiency while reducing costs are facilitators of successful implementation and adoption in clinical settings [7].

Evidence on AI implementation in clinical settings is growing rapidly; however, a synthesis of the evidence, and specifically a synthesis grounded in implementation science frameworks and theories, has not been conducted. Implementation science, which is defined as the systematic study of how evidence-based practices are feasibly adopted, implemented, and sustained in real-world settings [8,9], can play a crucial role in ensuring that AI can generate positive impacts in health care settings. Specifically, implementation science can support feasible, reliable, and equitable use of AI in health care settings, the critical step to generating impact, through an in-depth assessment of implementation determinants and the strategies that can be used to address or leverage these determinants [10-13]. By systematically organizing implementation determinants—ranging across individual health care providers, organizational structures, and the wider policy context—we can inform the development of tailored implementation strategies designed to support the effective and sustainable use of AI tools in clinical care [11,14]. A synthesis of AI implementation in clinical settings can also inform the conceptualization and categorization of determinants that may be unique to AI tools in clinical settings, further developing and refining implementation science frameworks and measures for the context of digital advancements in health care.

To address this knowledge gap and facilitate the transition of AI from experimental success to sustained and beneficial clinical impact, we conducted an umbrella review of reviews exploring determinants of AI implementation in clinical settings. The aim of this review was to qualitatively and comprehensively describe barriers and facilitators to AI implementation in clinical care settings. To do so, this review used the updated Consolidated Framework for Implementation Research (CFIR) to systematically catalog common barriers and facilitators identified in the literature [13]. While CFIR provides a comprehensive lens to examine the multilevel nature of implementation, we hypothesized that there may be unique determinants for AI. Therefore, we also sought to identify determinants unique to AI implementation that are not currently conceptualized and categorized within CFIR.

## Methods

### Study Design

This review is a subanalysis of a larger umbrella review examining barriers to and facilitators of digital health tool (DHT) implementation in clinical settings. This review and the parent review followed established methods for conducting scoping reviews [15,16] and the PRIOR (Preferred Reporting Items for Overviews of Reviews) reporting guideline (Checklist 1) [17]. The parent review protocol is registered with PROSPERO [18]. This review deviates from the registered protocol as this is a subanalysis of the parent review. An umbrella review is an appropriate method given the rapid pace of AI implementation, the volume of reviews on AI implementation, and the need to comprehensively synthesize implementation determinants for AI in clinical settings [19].

### Parent Review: Search Strategy

The parent umbrella review search strategy was conducted in 3 databases with the help of a reference librarian: PubMed, Scopus, and CINAHL (Multimedia Appendix 1). The initial search was conducted to identify all types of reviews (eg, systematic, scoping, and narrative) published in English between September 29, 2013, and November 6, 2023. These search dates are for the reviews and not the primary studies included in the reviews. An updated search was conducted to include reviews published through July 10, 2025. The date range was determined based on the time frame during which DHTs became more prevalent in health care [20]. The search terms included both MeSH and free-text terms related to digital tools, clinical care settings, and implementation (Multimedia Appendix 1).

### Parent Review: Inclusion and Exclusion Criteria

Reviews were included in the parent review if they met the following criteria: publication in English; description of determinants (barriers and facilitators) of implementing digital tools in clinical care settings (eg, inpatient hospital setting, outpatient routine care, and outpatient emergency care); publication between 2013 and 2025; and full-text availability. There was no geographic constraint. Reviews were included regardless of the geographic location in which the reviews’ included studies were conducted. Reviews were excluded if they focused on nonclinical settings, did not describe the implementation of digital tools, did not focus on health care providers (eg, physicians and nurses) as implementers of the DHTs, or did not provide detailed methods, such as search terms and dates and inclusion criteria, to be able to discern the origin of the results presented in each review (Multimedia Appendix 2).

### Parent Review: Screening

The initial search returned 4180 reviews. The updated search returned 1659 reviews, for a total of 5839 reviews. Reviews were imported into Covidence (Veritas Health Innovation), where duplicates were removed. For title and abstract screening and full text review, blinded dual independent screening was conducted. Consensus meetings were held regularly throughout screening to resolve conflicts or disagreements for inclusion (research team members: A Gilbert, A Gacad, LP, MKK, DT, and OE).

### Parent Review: Data Extraction

The study team collectively developed and pilot tested 2 data extraction protocols. The first protocol abstracted review characteristic data from all reviews in the parent review sample and was used to identify subsets of reviews to explore implementation determinants with a second data extraction protocol. This was done due to the large number of included reviews and time and resource intensity to qualitatively abstract implementation determinants. This paper focuses on the subset review for AI DHTs. Data from both extraction protocols were abstracted using dual-blinded independent review with consensus meetings.

In the first extraction protocol, data included the type of DHT (eg, AI, telehealth, and clinical decision support tools); the health care setting the review focused on (eg, outpatient routine care, outpatient emergency care, and inpatient); health or health care outcomes the review focused on (eg, chronic disease prevention and management, patient engagement and satisfaction, and health care use); and the geographic setting the review focused on (eg, United States only, countries outside the United States, and global [United States and other countries]). AI tools were defined as any DHT that used AI or ML, which may have overlapped with other DHT types (eg, telehealth and clinical decision support tools). If no data existed, reviewers marked “none specified.” Data were extracted by reviewers in Covidence (research team members: A Gilbert, A Gacad, LP, MKK, and DT).

### Subanalysis Review: Data Extraction

The second protocol was informed by CFIR, which outlines determinants of implementation across outer setting (eg, policies and law and partnerships), inner setting (eg, communications and available resources), implementer (eg, health care provider motivation and capability), and innovation (eg, AI complexity and design) levels. If a determinant did not fit within an existing construct, reviewers placed the text into “other” categories within the appropriate level (outer setting, inner setting, implementer, and innovation). If it was unclear whether a determinant was a barrier or facilitator, reviewers indicated it as both. Determinants were also indicated as both a barrier and a facilitator if the review presented them that way. Data were also extracted on whether a review used a theory, model, or framework (yes or no); the name of the theory, model, or framework; and how it was applied (eg, to inform research questions or aims, to support data extraction or analysis, and to interpret results or frame discussion).

The extraction template was built in Microsoft Excel and Qualtrics XM. Data extraction was conducted for all reviews identified through the first data extraction protocol that focused on AI DHTs. Extraction was conducted independently by 2 reviewers who copied and pasted review text into the Excel file (research team members: A Gilbert, A Gacad, LP, MKK, DT, ON, KT, WLT, BW, and PJ). Consensus meetings were held by the paired reviewers to discuss extraction and come to consensus for the final data. The final data were then entered into the Qualtrics form.

### Subanalysis Review: Data Synthesis

Data were downloaded from Qualtrics. Study team members (A Gilbert, A Gacad, MKK, and LP) reviewed and summarized the results for each CFIR construct and the “other” category. Study team members met to discuss the summarized results and emergent themes. The results are presented by CFIR domain or level by summarizing barriers and facilitators across the constructs using narrative synthesis.

### Subanalysis Review: Quality Assessment

The quality of reviews in this AI subanalysis review was assessed using the Joanna Briggs Institute (JBI) Critical Appraisal Checklist for Systematic Reviews and Research Syntheses through dual-blinded independent assessment and consensus meetings (research team members: A Gilbert, A Gacad, LP, and MKK) [21].

### Subanalysis Review: Primary Study Overlap Assessment

The study team used the Excel-based GROOVE (Graphical Representation of Overlap for Overviews) tool to calculate the corrected covered area (CCA) for reviews in this AI subanalysis review. The CCA is the prevalence of primary studies included in more than one of the included reviews [22]. The tool calculates the CCA for the sample, with a CCA of 0% to 5% representing a slight overlap, 6% to 10% a moderate overlap, 11% to 15% a high overlap, and above 15% a very high overlap.

### Subanalysis Review: Data Analysis

Data extracted from the reviews included in this subanalysis were downloaded from Qualtrics into an Excel file for analysis. All text that was deductively extracted into CFIR constructs was summarized through consensus meetings. The “other” categories for each level were analyzed using an inductive approach to identify themes within CFIR domains (innovation, implementer, inner setting, and outer setting) that did not fit within existing CFIR constructs. Inductive analyses were conducted using dual-blinded independent review with consensus meetings (research team members: A Gilbert and MKK). These themes are referred to as emergent themes in the *Results* section. We calculated the number of reviews from which data were extracted for each CFIR construct.

## Results

### Overview

The PRISMA (Preferred Reporting Items for Systematic Reviews and Meta-Analyses) flow diagram (Figure 1) shows the number of reviews at each stage of the review.

A total of 22 reviews were included in this AI subanalysis review, with publication years from 2021 to 2025 and covering studies conducted between 1990 and 2025 (Table 1). Reviews varied in type, including systematic reviews (n=16, 73%) [1-3,6,7,23-33], scoping reviews (n=2, 9%) [34,35], rapid reviews (n=1, 5%) [36], literature reviews (n=1, 5%) [37], and narrative reviews (n=2, 9%) [4,38]. Most reviews (n=18, 82%) [1,3,4,6,7,23-30,32,34-37] did not specify a geographic region, although several (n=3, 14%) [2,31,38] reported focusing solely on non-US contexts. Eight (36%) reviews focused on specific health care settings, including inpatient settings (n=4, 18%) [25,27,31,36], outpatient routine care (n=5, 23%) [27,29,31-33], and outpatient emergency care (n=3, 14%) [25,27,30]. Eleven (50%) reviews applied one or more theories, models, or frameworks (TMFs) [1,2,4,6,23,24,27,29,32,33,35]. One (5%) review used TMFs to inform the research question or aim [35], 9 (41%) reviews used TMFs to support data extraction or analysis [1,2,4,24,27,29,32,33,35], and 6 (27%) used TMFs to interpret results and frame the discussion [1,2,6,29,33,35].

Nine (41%) reviews had a quality assessment of 100% [1-3,6,24,25,29,30,32], 14 (64%) reviews had a quality assessment of 80% or higher [1-4,6,23-25,27,29,30,32,33,38], and 4 (18%) reviews had a quality assessment of 50% or less (Table 2) [7,26,31,36]. The CCA for the included reviews indicated slight overlap at 0.4%. Of the 706 primary studies, 655 (92.8%) studies were only included in 1 review, 43 (6.1%) studies were included in 2 reviews, and 2 (0.3%) studies were included in 3 reviews.

**Figure 1.**
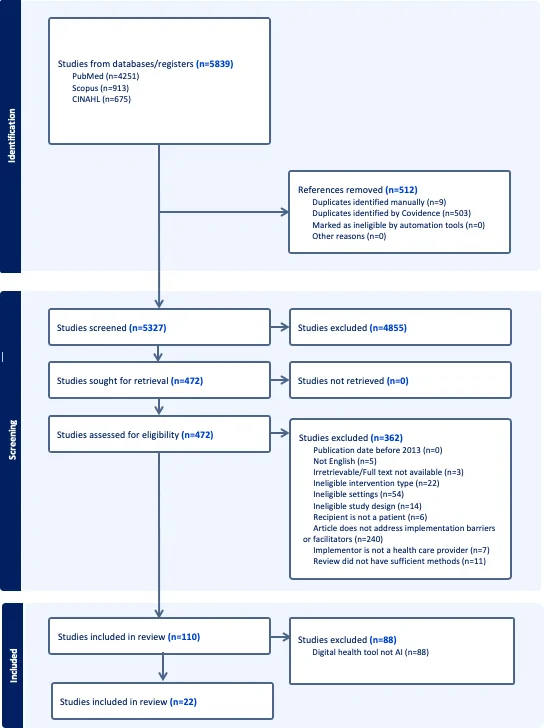
PRISMA (Preferred Reporting Items for Systematic Reviews and Meta-Analyses) flow diagram of review selection for the AI digital health tool subanalysis.

**Table 1.** Review characteristics of the 22 reviews included in the AI digital health tool subanalysis.

Author, year	Title	Publication years of primary articles	Primary articles, n	Type of review	Geographic context	Health care setting	Theory, model, or framework
Tossaint-Schoenmakers et al, 2021 [23]	The challenge of integrating eHealth into health care: systematic literature review of the Donabedian model of structure, process, and outcome	2012‐2019	11	Systematic review	Not specified	Not specified	Donabedian framework of structure-process-outcome [39]
Clement and Maldonado, 2021 [36]	Augmenting the transplant team with artificial intelligence: toward meaningful AI use in solid organ transplant	2020‐2021	74	Rapid review	Not specified	Inpatient settings	—^a^
van der Vegt et al, 2023 [24]	Deployment of machine learning algorithms to predict sepsis: systematic review and application of the SALIENT clinical AI implementation framework	2015‐2022	30	Systematic review	Not specified	Not specified	SALIENT implementation framework [40]
Eltawil et al, 2023 [34]	Analyzing barriers and enablers for the acceptance of artificial intelligence innovations into radiology practice: a scoping review	2013‐2022	12	Scoping review	Not specified	Not specified	—
Kamel Rahimi et al, 2024 [25]	Implementing AI in hospitals to achieve a learning health system: systematic review of current enablers and barriers	2019‐2022	26	Systematic review	Not specified	Inpatient settings; outpatient emergency care	—
Wubineh et al, 2024 [26]	Exploring the opportunities and challenges of implementing artificial intelligence in healthcare: a systematic literature review	2016‐2022	33	Systematic review	Not specified	Not specified	—
Preti et al, 2024 [27]	Implementation of machine learning applications in health care organizations: systematic review of empirical studies	2015‐2023	34	Systematic review	Not specified	Inpatient settings; outpatient routine care; outpatient emergency care	Consolidated Framework for Implementation Research [13]
Ayorinde et al, 2024 [4]	Health care professionals’ experience of using AI: systematic review with narrative synthesis	1990‐2023	25	Narrative review	Not specified	Not specified	Meta-UTAUT [41]
Wah, 2025 [28]	Revolutionizing e-health: the transformative role of AI-powered hybrid chatbots in healthcare solutions	2022‐2025	29	Systematic review	Not specified	Not specified	—
Busch et al, 2025 [6]	Current applications and challenges in large language models for patient care: a systematic review	2022‐2023	89	Systematic review	Not specified	Not specified	Unnamed [6]
Aravazhi et al, 2025 [7]	The integration of artificial intelligence into clinical medicine: trends, challenges, and future directions	2014‐2024	8	Systematic review	Not specified	Not specified	—
Qi et al, 2025 [2]	Utilization of artificial intelligence in clinical practice: a systematic review of China’s experiences	2020‐2024	28	Systematic review	Countries outside the United States	Not specified	Nonadoption, abandonment, scale-up, spread, and sustainability framework [42]
Goh et al, 2025 [29]	Challenges in implementing artificial intelligence in breast cancer screening programs: systematic review and framework for safe adoption	2019‐2023	20	Systematic review	Not specified	Outpatient routine care	Consolidated Framework for Implementation Research; AI governance framework [29]
El Arab and Al Moosa, 2025 [30]	The role of AI in emergency department triage: an integrative systematic review	2018‐2024	26	Systematic review	Not specified	Outpatient emergency care	—
Aljehani and Al Nawees, 2025 [31]	The current state, challenges, and future directions of artificial intelligence in healthcare in Saudi Arabia: systematic review	2018‐2023	13	Systematic review	Countries outside the United States	Inpatient settings; outpatient routine care	—
Arvai et al, 2025 [35]	Health care professionals’ concerns about medical AI and psychological barriers and strategies for successful implementation: scoping review	2016‐2023	32	Scoping review	Not specified	Not specified	Cognitive dissonance theory [43]; protection motivation theory [44]; attitude scale [35]
Jacob et al, 2025 [1]	AI for IMPACTS framework for evaluating the long-term real-world impacts of AI-powered clinician tools: systematic review and narrative synthesis	2019‐2024	44	Systematic review	Not specified	Not specified	Sociotechnical theory [45]; AI for IMPACTS framework [1]
Sahoo et al, 2025 [3]	Health professionals’ perspectives on the use of artificial intelligence in healthcare: a systematic review	2019‐2022	11	Systematic review	Not specified	Not specified	—
Abdelaziz et al, 2025 [37]	Perceptions and attitudes of registered nurses and nursing students toward advanced technology and artificial intelligence: a review of literature	2018‐2022	10	Literature review	Not specified	Not specified	—
Yousefi et al, 2025 [32]	Opportunities, challenges, and requirements for artificial intelligence (AI) implementation in primary health care (PHC): a systematic review	2009‐2024	109	Systematic review	Not specified	Outpatient routine care	Primary care evaluation tool [46]
Abdulazeem et al, 2025 [33]	Knowledge, attitude, and practice of primary care physicians toward clinical AI-assisted digital health technologies: systematic review and meta-analysis	2018‐2024	24	Systematic review	Global	Outpatient routine care	IDEAS framework [47]
Mwogosi et al, 2025 [38]	AI-IoT integration in Tanzania’s primary healthcare system: a narrative review	2015‐2024	21	Narrative review	Countries outside the United States	Not specified	—

a Not applicable.

**Table 2.** Joanna Briggs Inventory (JBI) Quality assessment for 22 reviews included in the AI digital health tool subanalysis.

Review	Quality assessment^a^	Is the review question clearly and explicitly stated?	Were the inclusion criteria appropriate for the review question?	Was the search strategy appropriate?	Were the sources and resources used to search for studies adequate?	Were the criteria for appraising studies appropriate?	Was critical appraisal conducted by 2 or more reviewers independently?	Were there methods to minimize errors in data extraction?	Were the methods used to combine studies appropriate?	Was the likelihood of publication bias assessed?	Were recommendations for policy and/or practice supported by the reported data?	Were the specific directives for new research appropriate?
Abdelaziz et al, 2025 [37]	60%	Yes	Yes	Yes	Yes	No	No	No	Unclear	N/A^b^	Yes	Yes
Abdulazeem et al, 2025 [33]	91%	Unclear	Yes	Yes	Yes	Yes	Yes	Yes	Yes	Yes	Yes	Yes
Aljehani and Al Nawees, 2025 [31]	50%	Yes	Unclear	No	Yes	Yes	Unclear	No	Unclear	N/A	Yes	Yes
Aravazhi et al, 2025 [7]	50%	Yes	Yes	No	Yes	No	No	No	Unclear	N/A	Yes	Yes
Arvai et al, 2025 [35]	60%	Yes	Yes	Yes	No	Unclear	Yes	No	Unclear	N/A	Yes	Yes
Ayorinde et al, 2024 [4]	90%	Unclear	Yes	Yes	Yes	Yes	Yes	Yes	Yes	N/A	Yes	Yes
Busch et al, 2025 [6]	100%	Yes	Yes	Yes	Yes	Yes	Yes	Yes	Yes	N/A	Yes	Yes
Clement and Maldonado, 2021 [36]	50%	Yes	Unclear	Yes	Yes	No	No	No	No	N/A	Yes	Yes
El Arab and Al Moosa, 2025 [30]	100%	Yes	Yes	Yes	Yes	Yes	Yes	Yes	Yes	N/A	Yes	Yes
Eltawil et al, 2023 [34]	60%	Yes	Yes	Yes	Yes	No	No	No	Unclear	N/A	Yes	Yes
Goh et al, 2025 [29]	100%	Yes	Yes	Yes	Yes	Yes	Yes	Yes	Yes	N/A	Yes	Yes
Jacob et al, 2025 [1]	100%	Yes	Yes	Yes	Yes	Yes	Yes	Yes	Yes	N/A	Yes	Yes
Kamel Rahimi et al, 2024 [25]	100%	Yes	Yes	Yes	Yes	Yes	Yes	Yes	Yes	N/A	Yes	Yes
Mwogosi et al, 2025 [38]	80%	Yes	Yes	Yes	Yes	Unclear	Unclear	Yes	Yes	N/A	Yes	Yes
Preti et al, 2024 [27]	80%	Yes	Yes	Yes	Yes	Yes	Unclear	Unclear	Yes	N/A	Yes	Yes
Qi et al, 2025 [2]	100%	Yes	Yes	Yes	Yes	Yes	Yes	Yes	Yes	Yes	Yes	Yes
Sahoo et al, 2025 [3]	100%	Yes	Yes	Yes	Yes	Yes	Yes	Yes	Yes	N/A	Yes	Yes
Tossaint-Schoenmakers et al, 2021 [23]	90%	Yes	Yes	Yes	Yes	Yes	Unclear	Yes	Yes	N/A	Yes	Yes
van der Vegt et al, 2023 [24]	100%	Yes	Yes	Yes	Yes	Yes	Yes	Yes	Yes	N/A	Yes	Yes
Wah, 2025 [28]	60%	Yes	Yes	Unclear	Yes	No	No	No	Yes	N/A	Yes	Yes
Wubineh et al, 2024 [26]	50%	Yes	Yes	No	Yes	No	Unclear	Unclear	Unclear	N/A	Yes	Yes
Yousefi et al, 2025 [32]	100%	Yes	Yes	Yes	Yes	Yes	Yes	Yes	Yes	Yes	Yes	Yes

a Overall quality assessment percentage based on number of “yes” over the 11 JBI questions that were applicable for the review.

b N/A: not applicable.

### Determinants of AI Implementation Mapped to CFIR

#### Innovation Determinants

Across reviews, the most frequently identified innovation-level determinants included the *evidence base* (barriers: n=11 reviews [2-4,6,24,25,29,32,35,36,38] and facilitators: n=7 reviews [4,23-25,32,37,38]), *relative advantage* (barriers: n=13 reviews [1-4,24,25,27-29,32,35,36,38] and facilitators: n=14 reviews [2-4,24,27,30-35,37,38,48]), and *design* (barriers: n=13 reviews [1,2,4,6,7,23-29,38] and facilitators: n=8 reviews [1,2,7,24,25,27,37,38]; Table 3). The *evidence base* for AI lacks sufficient or reliable data on clinical impact to support AI adoption and implementation. However, evidence of clinical impact, especially if locally relevant (ie, to a specific health care setting, disease, and population), was reported as a potential support for AI implementation. AI was described as having a *relative advantage* because of its high potential to improve clinical decision-making, efficiency, and patient care and to reduce provider burden and costs. However, the relative advantage of AI was noted as having limited impact on facilitating implementation, given issues with *adaptability (barriers*: n=12 reviews [1,2,4,6,23,25,27,28,35-38] and *facilitators*: n=6 reviews [1,2,25,27,37,38]) and *complexity* (barriers: n=8 reviews [1,2,4,23,28,30,35,38] and facilitators: n=4 reviews [2,25,30,37]). Reviews indicated that barriers to using AI in clinical practice were poor interpretability and lack of applicability to specific diseases, populations, task types, or clinical settings and workflows. *Design*-related barriers included a lack of high-quality and representative data; poor model upkeep, which reduced the reliability and validity of AI output; and interfaces that were not easy for providers to use. Co-designing AI with health care providers was described as a facilitator of AI implementation. *Cost* (barriers: n=7 reviews [1,6,23,28,32,35,37] and facilitators: n=4 reviews [1,2,23,38]) and *source* (barriers: n=5 reviews [7,24,29,33,35] and facilitators; n=3 reviews [23,33,35]) were less frequently cited determinants. Reviews noted that adopting AI and integrating AI into existing systems could be costly; however, the potential benefits of reduced health care expenditure resulting from AI tools may provide financial benefits. In general, the source (ie, AI model developers) of the AI tool was described as important for ensuring the AI model met health care needs, fit into workflows, and could be easily used by providers. Trialability was not presented by reviews as a determinant for AI implementation.

Emergent innovation-level constructs included *data input and quality*, *transparency*, *accountability*, *reliability*, *validity*, *model maintenance*, *model bias*, *robustness*, and *generalizability* (Table 4). The unique intersection around the lack of *transparency* for how an AI tool arrives at a decision; the limitations for providers to determine the *validity* of the AI output; be able to *rely* on the model output across clinical encounters; and the need to continually monitor, evaluate, and maintain AI models hinted at determinants not fully captured through CFIR constructs (ie, design, evidence base, and complexity). The quality and representativeness of the *data input* for the model and consequently the model’s *robustness, generalizability*, and *biases*; and therefore its utility across populations and settings were described in reviews as critical for model uptake, scalability, and reaping the potential benefits of AI tools in clinical practice.

**Table 3.** CFIR^a^ constructs described as determinants for AI digital health tool subanalysis (n=22)^b^.

CFIR construct	Barrier		Facilitator	
	Reviews, n	Quote	Reviews, n	Quote
Innovation				
Source	5	“Inexperienced specialists in AI development” (Arvai et al [35]).	3	“HCPs^c^ should be involved in AI development for medicine and HC^d^” (Arvai et al [35]).
Evidence base	11	“Lack of relevant empirical evidence on AI clinical performance and of its impact on healthcare workflow and quality” (Ayorinde et al [4]).	7	“Track record of healthcare professionals’ action, based on AI recommendation and the corresponding patient outcomes could facilitate confidence in AI tools” (Ayorinde et al [4]).
Relative advantage	13	“A key limitation was that many AI tools were designed for narrow tasks and were not suitable for comprehensive clinical decision-making. This constrained their perceived utility among clinicians, who continued to rely on human judgment for decisions” (Qi et al [2]).	14	“General Physicians (GP) also believe that using AI tools can save their time for activities that require a high level of licensure and reduce time-consuming tasks. AI can also assist with charts, documentation, and data entry, allowing providers to think critically and treat patients during scheduled visits” (Sahoo et al [3]).
Adaptability	12	“Not adapted to local workflows or different clinicians’ workflows; perceived poor ability of the application to take contextual factors” (Preti et al [27]).	6	“Adapting systems to local context of implementation; training model on cases in local context” (Preti et al [27]).
Complexity	8	“AI being perceived as too complicated” (Arvai et al [35]).	4	“Several teams employed SHapley Additive exPlanations (SHAP) or rule-based decision trees to enhance interpretability” (El Arab and Al Moosa [30]).
Design	13	“Lack of integration of disparate data sources; data set shift; overfitting and underfitting of models; not updating the model with continuous data to retrain model; alert fatigue; reliance on third-party software” (Kamel Rahimi et al [25]).	8	“Co-designing solutions with end-user input ensure relevance, usability, and alignment” (Mwogosi et al [38]).
Cost	7	“Concerns were raised regarding the economic burden of adopting AI technologies (10.09%) and financial uncertainties and barriers (5.50%). These challenges primarily stem from the high costs of integrating advanced systems into existing infrastructures. Within the reimbursement structure, studies pointed to the complexity of AI reimbursement processes (6.42%) as a critical issue” (Yousefi et al [32]).	4	“Several studies reported that AI implementation reduced direct healthcare expenditures, including testing fees, registration costs, and staffing needs in automation and triage support” (Qi et al [2]).
Implementer				
Capability	15	“Frontline clinicians in primary care and junior medical staff often expressed lower confidence in AI tools due to limited exposure or prior experience” (Qi et al [2]).	9	“Intermediate and advanced knowledge levels might enhance the adoption of AI in clinical practice” (Arvai et al [35]).
Opportunity	6	“the anticipated cognitive and administrative workload of learning AI concepts and the complexity of AI troubleshooting compete with the prioritized immediate clinical responsibilities” (Abdulazeem et al [33]).	2	“Collaborations between the government, private sector, and non-governmental organizations can mobilize resources and expertise for implementing AI-IoT^e^ projects” (Mwogosi et al [38]).
Inner setting				
Structural characteristics	11	“Technical readiness varied across institutions. While large hospitals demonstrated the infrastructure and human resources to support AI deployment, smaller hospitals and rural clinics often lacked IT systems, network capacity, or technical expertise. Second, unclear internal governance structures weakened institutional capacity for scale-up. In some settings, hospitals lacked formal workflows for AI evaluation, making to fragmented implementation and reduced accountability. Third, resource-limited facilities expressed concern about the sustainability of AI systems, particularly when deployment lacked training or support. In such settings, the absence of continuous professional development led to inconsistent follow-up into daily workflow” (Qi et al [2]).	9	“Infrastructure plays a crucial role in the successful implementation of AI tools in health care settings. Adequate computational power, specialized hardware, and robust IT infrastructure are often necessary to support the processing of large datasets and the operational demands of AI technologies” (Jacob et al [1]).
Culture	3	“The specific collection of values and norms that are shared by people within an organization and the internal collaboration and collective engagement.” (Tossaint-Schoenmakers et al [23]).	1	“Institutions that fostered a learning-oriented culture that support innovation were more open to piloting AI tools and refining their implementation.” (Qi et al [2]).
Tension for change	1	“These facilities, however, face systemic inefficiencies that hinder effective service delivery. Delays in data processing, resource mismanagement, high patient loads, and the lack of decision-support tools are common challenges that strain healthcare providers’ capacity” (Mwogosi et al [38]).	1	“High clinician workload and job complexity made these candidates for AI uptake” (Kamel Rahimi et al [25]).
Compatibility	12	“Poor integration or failure to align with clinical routines can negatively impact efficiency, increase cognitive burdens, and require significant resources to adapt systems” (Jacob et al [1]).	9	“Scalability was further supported when AI systems aligned with existing hospital workflows or could be built upon current platforms, such as EMRs^f^ or remote monitoring systems” (Qi et al [2]).
Incentive systems	3	“Developing comprehensive incentive structures (3.67%) was noted as an important step in motivating stakeholders and aligning their goals with AI adoption strategies” (Yousefi et al [32]).	3	“Finally, within the reimbursement structure, studies highlighted the necessity of transforming healthcare reimbursement models and insurance coverage (1.83%) to accommodate AI-driven” (Yousefi et al [32]).
Mission alignment	0	—^g^	2	“Strong support from leadership” (van der Vegt et al [24]).
Available resources	10	“High costs associated with AI equipment installation and management” (Arvai et al [35]).	5	“Personnel time for integration of new ML^h^ system” (Kamel Rahimi et al [25]).
Access to knowledge and information	12	“The lack of adequate training and knowledge of these technologies and the ethics behind their use of them leads to skepticism and a reluctance to adopt these new changes” (Abdelaziz et al [37]).	10	“Accessible and responsive technical support is necessary to address user concerns, provide ongoing assistance, and maintain confidence in the AI tool’s reliability and safety over time” (Jacob et al [1]).
Outer setting				
Critical incidents	0	—	2	“National policy initiatives played a role in facilitating AI adoption. Government funding programs provided both financial and strategic support.33,34,46,50 This was particularly evident during the COVID-19 pandemic, when the urgency of the crisis accelerated the deployment of digital tools” (Qi et al [2]).
Local attitudes	4	“Healthcare professionals were concerned patients may not be willing to have AI used for them” (Ayorinde et al [4]).	0	—
Local conditions	5	“Regional differences in digital infrastructure and digital literacy at population level created uneven conditions for implementation in resource-limited areas” (Qi et al [2]).	4	“Expanding AI availability in rural healthcare settings (6.42%) and balancing geographical access and distribution of health technologies (4.59%) were highlighted as essential” (Yousefi et al [32]).
Partnerships and connections	3	“Limited stakeholders’ collaboration” (Yousefi et al [32]).	8	“Establishing cross-disciplinary collaboration between AI developers, healthcare professionals, and stakeholders can mitigate resistance to AI adoption in healthcare” (Abdulazeem et al [33]).
Policies and laws	13	“Healthcare professionals expressed concerns over regulatory and legal uncertainties surrounding AI use” (Ayorinde et al [4]).	6	“Enabling environments shaped by policy, governance, and institutional commitment” (Qi et al [2]).
Funding	5	“Insufficient capital investment” (Aljehani and Al Nawees [31]).	2	“Within healthcare expenditures, fostering strategic investment and financial (11.01%) was identified as a critical requirement for enabling a sustainable environment for AI adoption in PHC^i^. Additionally, assessing the economic viability and cost-effectiveness of AI implementation (7.34%) was emphasized as a key step to ensuring affordability and feasibility” (Yousefi et al [32]).

a CFIR: Consolidated Framework for Implementation Research.

b Constructs not identified in any review: innovation, trialability; implementer, need and motivation; inner setting, relational connections, communications, and relative priority; outer setting, external pressure.

c HCP: health care professional.

d HC: health care.

e AI-IoT: AI–Internet of Things.

f EMR: electronic medical record.

g Not applicable.

h ML: machine learning.

i PHC: primary health care.

**Table 4.** Emergent constructs for AI digital health tool subanalysis (n=22)^a^.

Constructs	Definition	Quote
Innovation		
Data inputs and quality	Data used for training and testing the AI including data volume, quality, and missing values or completeness, representativeness of sampling, and use of local data	“Data quality and missing values often limited immediate adoption” (El Arab and Al Moosa [30]). “Representative patient data for training, testing, and validation of AI algorithms” (Abdulazeem et al [33]). “Key data such as ethnicity (reported in only 3.6% of approvals), socioeconomic information (absent in 99.1%), and study participants’ age (missing in 81.6%) were often underreported” (Jacob et al [1]). “Users concern about the potential for AI to worsen inequity as disadvantaged populations are not represented in health data sets” (Ayorinde et al [4]).
Transparency	Black box nature of algorithms and clarity around how AI models arrive at their conclusions	“A major limitation was called “black box” nature of many AI algorithms, which lacked transparency, thereby making clinician skepticism and reducing trust. In such systems, the decision-making process was often opaque, making it difficult for users to understand how inputs were translated into recommendations. This lack of transparency not only limited accountability but also raised concerns regarding diagnostic accuracy, and the ability to validate results in complex or high-risk cases” (Qi et al [2]). “The tendency of AI to recommend individualized decisions that are not explainable, rather than providing general recommendations at a population level, as in a conventional screening setting. The term “black box” refers to an AI system whose internal workings are not transparent or easily understandable. Intellectual property clauses protecting proprietary sources fuel the lack of transparency by limiting the distribution and sharing of the AI source code and architecture. With limited human involvement in the decision-making process and a lack of clear explanation for how AI systems arrive at their recommendations, there is significant skepticism and reduced trust in the decisions made with the help of AI” (Goh et al [29]). “Lack of clarity and transparency in AI decision-making processes” (Yousefi et al [32]). “The opaque nature of AI algorithms” (Arvai et al [35]). “Explainability and interpretability are essential for ensuring the ethical and trustworthy use of AI tools in health care. These criteria allow health care professionals to understand how AI models arrive at their conclusions, fostering trust in their recommendations. Explainability helps to demystify the AI’s decision-making process, making it transparent and accessible to users. This, in turn, improves adoption, as clinicians are more likely to trust and rely on AI tools that are interpretable. Ultimately, clear explainability supports ethical deployment, reducing risks associated with “black box” systems” (Jacob et al [1]).
Reliability	Consistency of AI output	“Large variability could affect the perceived clinical applicability of AI-generated risk assessments” (Goh et al [29]). “Concerns about clinical reliability” (El Arab and Al Moosa [30]). “There were also worries about the unpredictability of AI results and the potential for errors in clinical AI” (Arvai et al [35]). “The reliability of the AI model after its implementation, ensuring it consistently avoids errors and unintended consequences” (Jacob et al [1]).
Validity	Accuracy of AI output	“Doubts about the accuracy of AI” (Arvai et al [35]). “GPs^b^ were worried that AI assistants might make errors, limiting their benefits if excessive time was spent revising them, the majority of GPs voiced apprehensions about AI’s capability to handle clinical reasoning and diagnostic decisions in general practice, advocating for AI assistance in resolving clinical uncertainties but not making final decisions” (Sahoo et al [3]).
Model maintenance	Ongoing evaluation and upkeep of the model	“Ongoing model maintenance” (El Arab and Al Moosa [30]). “The maintenance and updating of AI tools are critical to ensuring their continued effectiveness and safety in health care. Regular updates, including adjustments to algorithms and reference datasets, are essential to avoid performance degradation and ensure accurate results. Without proper maintenance, different software versions could introduce biases or inconsistencies, which might affect clinical outcomes” (Jacob et al [1]).
Model bias	Model accuracy across different groups.	“Risk of algorithmic biases” (Abdulazeem et al [33]). “The risk of health inequalities, such as bias toward light skin types in algorithm training data” (Arvai et al [35]). “The risk of bias in training data, including sample size and representativeness, which can lead to unequal treatment or outcomes for underrepresented populations” (Jacob et al [1]).
Robustness	Ability to perform consistently or accurately across different populations, complex scenarios, unexpected situations, diverse groups or population, and complex conditions	“There were concerns about AI tool not being able to account for factors such as patient’s culture and ground context and therefore limiting the potential impact of the AI” (Ayorinde et al [4]). “The poor performance of AI in unexpected situations” (Arvai et al [35]). “Limitations on the ability of AI platforms to handle complex cases were some of the concerns raised by physicians” (Sahoo et al [3]). “Ensures the tool can maintain high performance even when exposed to slight variations in input data or operational environments” (Jacob et al [1]). “Greater condition complexity could reduce the clinical appropriateness of AI applications and restrict their effectiveness. Such complexity, as well as diagnostic subjectivity, frequently contributed to implementation barriers. AI struggled to adapt to multifactorial conditions involving complications, such as cancer, skeletal disorders, or viral hepatitis with etiologies, where expert judgment remained indispensable. Additionally, conditions that required symptom interpretation, such as asthma exacerbation, unexplained abdominal pain, or psychiatric symptoms, often exceeded the interpretative capacity of AI systems, which limited their perceived clinical value. Beyond clinical complexity, patient engagement and disease awareness also played a critical role in determining the suitability of AI applications. In cases where users had poor understanding of their condition, incomplete data inputs diminished model performance and intervention efficacy” (Qi et al [2]).
Generalizability	Model performs in environments, for conditions and populations it was not trained for	“Evaluates whether the AI tool can effectively perform across different populations, clinical settings, or geographic regions beyond the environment in which it was trained” (Jacob et al [1]).
Implementer		
Trust	Providers can rely on AI output for accurate and consistent information	“Trust and transparency emerged as the second most significant, emphasizing deficits in trust and confidence” (Yousefi et al [32]). “The acceptance of AI largely depends on physicians’ trust in AI-generated information, particularly in diagnostic accuracy” (Abdulazeem et al [33]). “Trust is built through factors such as validation, transparency, safety, privacy, and interpretability of the AI tool. Both health care professionals and patients must trust that the AI tool is reliable, safe, and effective in clinical practice” (Jacob et al [1]).
Human element	Model is co-designed with providers and populations impacted by the use of the AI model Communication, empathy, and alignment with personalized goals in the experience of care delivery	“Patient-centeredness/ Doctor-patient relationship” (Abdulazeem et al [33]). “Its lack of empathy or communication” (Arvai et al [35]). “Alignment with personalized care goals” (Qi et al [2]). “Concerns have been raised that the increasing reliance on AI may undermine essential human elements in health care, such as nuanced clinical judgment and collaborative care, potentially affecting the quality of patient experience and outcomes” (Goh et al [29]).
Existential threat	Provider experiences related to fear, anxiety and potential loss of skills, job, and importance due to the AI model	“Risks of over-reliance on AI” (Yousefi et al [32]). “Fears of skill degradation and professional displacement among experienced practitioners. Emotional and psychological factors such as anxiety and a perceived lack of control further discouraged adoption, even when the perceived utility was high” (Qi et al [2]). “Concerns were also raised regarding the broader impact of AI on professional development and society. There are fears that AI may lead to job displacement or increased dependency among radiologists, particularly if it assumes responsibility for interpreting normal mammograms. Similarly, Carter et al and Hickman et al warned that overreliance on AI in such tasks could diminish radiologists’ diagnostic skills or reduce their familiarity with normal imaging, potentially resulting in the oversight of important clinical nuances” (Goh et al [29]). “Optimism fluctuates because of concerns about AI surpassing physicians’ diagnostic capabilities and the fear of being replaced. Rony and colleagues found that some healthcare workers fear job replacement and expressed concerns about AI’s capabilities and the hazard of being technologically unemployed. Therefore, concerns over job security remain critical, as reliance on AI could eventually diminish decision-making skills and raise concerns regarding clinical autonomy” (Abdulazeem et al [33]). “The fear of being replaced” (Arvai et al [35]).
Bias	Providers choose not to use AI or to not use AI with certain populations out of concern for bias leading to differences in AI model reach and impact in certain populations Overreliance on AI leads to biased decision-making	“Some doctors expressed concerns about the potential development of diagnostic bias, wherein technology use may influence their judgment, potentially affecting the course and outcome of treatment” (Sahoo et al [3]). “Other potential biases include automation bias, where readers may be overly influenced by AI-generated decisions, leading to overcommitment to false positives or omission of other abnormalities” (Goh et al [29]). “Healthcare professionals may not actively consider their cases and they may not be able to refine their skills and maintain cultural competence” (Ayorinde et al [4]).
Relational component	Divergence in opinion between providers and AI recommendation creates uncertainty	“Divergence in opinion of healthcare professionals and AI recommendation creates uncertainty” (Ayorinde et al [4]). “Need for highly qualified practitioners to work alongside AI tools for optimal outcomes in in healthcare settings” (Sahoo et al [3]).
Accountability	Allocation of responsibility for AI decisions	“Implementation of AI in clinical settings often depended on unclear accountability for AI-driven decisions” (Qi et al [2]). “Another legal challenge is the determination of legal liability if AI systems in breast screening make errors leading to misdiagnosis or patient harm” (Goh et al [29]).
Inner setting		
Data security and privacy	Institution’s responsibility for protecting sensitive data processed by AI and managing liability in case of errors	“Ensuring compliance with relevant regulations, such as General Data Protection Regulation or Health Insurance Portability and Accountability Act, is essential to safeguard patient privacy. In addition, clear processes for obtaining informed consent are critical, ensuring that patients understand how their data will be used. Proper data ownership policies must also be in place, ensuring transparency around who controls the data and how it can be accessed or shared” (Jacob et al [1]). “Data breaches in the context of AI are particularly concerning, as they refer to incidents where unauthorized individuals or entities gain access to sensitive and confidential information processed or stored by AI systems. These breaches can have significant consequences, ranging from privacy violations to identity theft and other forms of cybercrime” (Goh et al [29]).
Ethics	Clinical decisions and differences in care provided resulting from AI model implementation can lead to poor health outcomes and health disparities and inequities Use of patient data and risk of data security and privacy breaches Inequities in care and health outcomes related to AI bias at the innovation and implementer level	“Persistent concerns regarding data security” (Abdulazeem et al [33]). “Issues of ethics and potential medico-legal concerns in relation to image manipulation and cybersecurity were also highlighted” (Arvai et al [35]). “Data security, privacy, informed consent, and data ownership are vital criteria for assessing AI tools in health care. These tools often require large amounts of sensitive patient data, which must be protected from unauthorized access, breaches, or misuse” (Jacob et al [1]). “The use of AI in healthcare raises issues with privacy and improper use of data” (Sahoo et al [3]).
Data availability and access	Institutions have data available to build, test, maintain, and evaluate the model	“Deficiencies in data quality and accessibility” (Yousefi et al [32]). “Professionals reported having access to more data about patient than AI algorithm uses” (Ayorinde et al [4]). “Poorer performance on patients who had less EHR^c^ data” (Kamel Rahimi et al [25]). “The limited availability of high-quality datasets for training and validation” (Arvai et al [35]).

a No emergent constructs were identified at the outer setting level.

b GP: general practitioner.

c EHR: electronic health record.

#### Implementer Determinants

Implementer *capability* (barriers: n=15 reviews [1-4,23-28,31-33,35,38] and facilitators: n=9 reviews [23-25,27,31,34,35,37,38]) was the most prominently reported implementer-level determinant for AI implementation (Table 3). *Capability* barriers included low confidence and limited experience using AI, particularly among frontline clinicians and newer medical staff. Providers lacked skills related to understanding AI output and AI rationale for decision-making. Higher AI literacy (ie, the ability to interact with AI) and higher levels of provider knowledge (ie, understanding of how AI works) facilitated AI implementation. *Opportunity* (barriers: n=6 reviews [4,23,32,33,35,38] and facilitators: n=2 reviews [4,38]) constraints described competing clinical demands and limited time available to learn or troubleshoot AI tools, which were exacerbated by the speed of AI technology advancements. *Motivation* and *need* were not presented as determinants for AI implementation.

Implementer-level emergent constructs included *trust*, *human element*, *existential threat*, *bias*, *relational component,* and *accountability*. These are unique determinants of AI that are not represented in implementation frameworks such as CFIR (Table 4). In particular, the threat to professional identity and potential loss of job, concerns of overreliance and skill loss, and lack of human connection within clinical care encounters point to specific implementation barriers that must be addressed for AI to be adopted. Reviews described how discrepancies between the clinical decisions of AI tools and those of providers could cause uncertainty in making clinical diagnoses and treatment decisions, limiting AI benefits for providers. These discrepancies also lead to concerns about accountability for clinical errors, misdiagnoses, and patient harm, reducing clinician uptake of AI tools. Model bias at the innovation level can also lead to bias at the provider level. Reviews highlighted how clinicians may choose not to use AI with certain populations out of concerns related to model bias, or, conversely, become overreliant on biased AI tools and lack the skills necessary for mitigating the real-world impact of biased AI output.

#### Inner Setting Determinants

Prominent inner setting determinants included *structural characteristics* (barriers: n=11 reviews [1,2,4,23,25,27,30,32,33,35,38] and facilitators: n=9 reviews [1,2,4,23,25,30,32,33,37]), *available resources* (barriers: n=10 reviews [2,23-26,28,30,31,35,38] and facilitators: n=5 reviews [1,25,30,31,38]), *access to knowledge and information* (barriers: n=12 reviews [1-4,7,24,29,32,33,35,37,38] and facilitators: n=10 reviews [1,2,4,24,25,27,31-33,37]), and *compatibility* (barriers: n=12 reviews [1,2,4,23,25,27,28,30,32,35,37,38] and facilitators: n=9 reviews [1,2,24,25,27,30,32,37,38]; Table 3). Reviews noted substantial variability in *structural characteristics*, *available resources, and access to knowledge and information*, with rural and smaller facilities experiencing limited technical infrastructure and resource constraints around IT, network connectivity, financial resources, and available training and expertise. Health care systems with robust IT infrastructure, access to technical support and ongoing training, and financial resources were better positioned to support AI implementation. Specifically, when considering *access to knowledge and information*, training to develop, operate, and maintain AI models; training to use and understand AI tools, including their limitations, implications, and the factors that might affect the performance of the AI tool; and technical support for troubleshooting were described as important for successful and sustained AI implementation. Reviews noted that other *structural characteristics,* such as lack of internal governance and formal workflows for AI integration, could hinder AI adoption. *Compatibility* facilitated implementation when there was integration of AI tools with existing systems, such as electronic medical records, and could limit AI implementation when the lack of integration into clinical workflow increased provider cognitive burden and disrupted clinical care. *Incentive systems* (barriers: n=3 reviews [23,32,33] and facilitators: n=3 reviews [2,32,33]), *culture* (barriers: n=3 reviews [23,27,38] and facilitators: n=1 review [2]), *tension for change* (barriers: n=1 review [38] and facilitators: n=1 review [25]), and *mission alignment* (barriers: n=0 reviews and facilitators: n=2 reviews [23,24]) were less commonly reported but were also determinants cited to influence implementation. Reviews noted that health care systems that fostered a learning culture, provided incentives through reimbursement models to use AI, and had leadership support were better positioned to adopt and implement AI tools. *Relational connections, communications*, and *relative priority* were not described by reviews as inner setting determinants for AI implementation.

Inner setting emergent constructs highlighted *data security and privacy*, *ethics*, *data availability and access*, and *monitoring and governance* as essential and unique determinants (Table 4). Inner setting governance and monitoring focus on institutional-level policies, formal workflows, and internal feedback mechanisms within health care organizations to ensure ethical compliance, protect data privacy, and safeguard health outcomes. In contrast, *outer setting policies and laws* refer to the broader national and regulatory frameworks, such as federal statutes (eg, Food and Drug Administration [FDA]) or state laws, that establish the legal environment for safety, security, and equity. While inner setting governance provides the specific operational oversight for an organization, outer setting policies address systemic issues such as liability and accountability to reduce the legal risks that often discourage AI adoption. For AI tools to be adopted, health care institutions must monitor and govern AI development and use through institutional policies, regulations, and feedback mechanisms that are aligned with federal, state, and other entities providing oversight (eg, professional associations). This oversight is necessary to protect data privacy, ensure compliance with ethical standards, and safeguard health and equity outcomes. These determinants were reported as salient for limiting negative ethical, legal, health, and equity consequences that could arise throughout the AI implementation process.

#### Outer Setting Determinants

The most common outer setting determinants included *policies and laws* (barriers: n=13 reviews [1-4,23,27-29,31-33,35,38] and facilitators: n=6 reviews [2,25,27,31,32,35]) and *partnerships and connections* (barriers: n=3 reviews [23,32,33] and facilitators: n=8 reviews [2,4,7,24,27,31-33]; Table 3). Reviews indicated that policies, laws, regulations, and guidelines were needed to ensure safety, data privacy and security, and equity and to address questions of liability and accountability for AI implementation. Specifically, reviews identified *policies and laws* primarily as a barrier, noting a lack of existing laws, regulations, and frameworks to support AI implementation. *Partnerships and connections* were more often described as facilitators of AI implementation. Reviews indicated that collaboration between health care providers, health care institutions, AI developers, regulatory officials, scientific societies, professional communities, and other stakeholders would facilitate integration, sustainability, and effectiveness of AI in clinical care settings. *Local attitudes* (barriers: n=4 reviews [2,4,28,38] and facilitators: n=0 reviews), *local conditions* (barriers: n=5 reviews [2,25,26,32,38] and facilitators: n=4 reviews [25,31,32,34]), and *funding* (barriers: n=5 reviews [23,28,31,32,38] and facilitators: n=2 reviews [2,32]) were generally described by reviews as barriers to AI implementation. These determinants related to available funding, network connectivity, and infrastructure within communities for supporting AI in health care systems, as well as provider perceptions about whether patients would be open to and comfortable with AI tools being used in their clinical care. *Critical incidents* (barriers: n=0 reviews and facilitators: n=2 reviews [2,31]), such as the COVID-19 pandemic, were described as facilitators for AI adoption. *External pressure* was not described as a determinant for AI implementation.

## Discussion

### Principal Findings

This umbrella review synthesized determinants of AI implementation in clinical settings across 22 reviews and revealed a complex, multilevel interplay of technical, individual, organizational, and policy-related factors. While enthusiasm for AI’s potential is high, persistent and interdependent barriers can hinder widespread implementation and sustained use. The findings illustrate that successful AI implementation requires addressing not only traditional determinants captured by CFIR but also numerous emergent constructs that reflect AI’s unique risks, complexities, and sociotechnical dimensions.

### Summary of Key Findings

This review identified determinants of AI implementation in clinical settings. Many of these determinants have not been observed in other forms of DHTs [49]. Specifically, key determinants included concerns about AI model development and data input as they relate to bias, equity, and validity; AI model transparency and upkeep for supporting clinician trust in AI tool accuracy and reliability; and usefulness of AI models for generalizability across conditions and populations.

These findings connect to the broader literature base, which shows that considerable progress has been made in developing *model interpretation* methods that aim to explain model predictions by quantifying the contributions of individual input variables at both the individual and population levels [50-53]. These methods are increasingly incorporated into clinical decision support tools to enhance clinicians’ understanding of model outputs and to surface factors associated with predicted risks [50-52]. However, it is important to recognize that most existing interpretation techniques characterize statistical associations rather than causal relationships. As a result, identified “important” features may reflect incidental or confounded correlations rather than true drivers of clinical outcomes. Substantial challenges, therefore, remain in advancing model interpretation approaches that can distinguish causal, actionable risk factors from spurious associations, which is essential for reliable clinical decision-making and effective intervention design [54]. In parallel with efforts to improve model interpretability, substantial progress has also been made in estimating and communicating the *uncertainty* associated with model outputs [55-58]. Explicitly reporting predictive uncertainty can help clinicians assess the reliability of AI-generated recommendations and calibrate their reliance on these outputs accordingly [59].

Our findings are aligned with other research, which shows that AI models often face substantial challenges in *generalizability and robustness* over time, as their performance can degrade when deployed in hospital systems or patient populations that differ from the data on which the models were trained. Recent efforts to address this issue include training models on large, diverse datasets that better represent broader populations, as well as developing foundation models pretrained on extensive clinical data to support a wide range of downstream tasks [60-63]. However, important questions remain regarding the extent to which even large-scale models can maintain robust performance in the presence of significant distribution shifts across institutions, care settings, and patient populations. These generalizability and robustness challenges have important *equity and fairness* implications [64,65]. Models trained on data that underrepresent certain demographic groups, socioeconomic contexts, or care settings may perform systematically worse for these populations [66,67], thereby exacerbating existing health disparities over the long term.

This review found that monitoring and governance are important determinants across multiple levels for AI adoption and implementation. Reviews underscored the need for regulations, laws, and policies at a variety of levels (federal vs state regulation, tort liability to clarify norms, contracting over the use of technologies, statutes governing the regulation of certain technologies through the FDA, professional society policies, and regulating the practice of medicine) and through different mechanisms to support responsible and successful AI adoption and address concerns about accountability, data privacy and security, and ethics.

The literature base suggests that some uses of AI in clinical practice can be governed or at least influenced by existing policies, such as some AI tools that are incorporated into medical devices and may be regulated federally by the FDA. Other uses of AI, such as ambient scribes, may fall outside of existing federal monitoring and governance frameworks, raising uncertainty about whether existing legal and regulatory frameworks, such as those governing health data privacy, sufficiently protect implementing facilities against legal risk. Even where some AI tools may be regulated for their safety and efficacy, other aspects of the same AI tool, such as allocation of liability, may lack clarity, such that the risk of liability may discourage adoption of the tool. For example, it may be unclear whether an AI developer or the adopting physician or health care facility would be the appropriate defendant in a case involving a patient harmed in the course of treatment involving an AI tool. The growing interest in how AI can be applied to clinical care has been accompanied by ongoing discussions on whether the legal accountability for decisions made falls on only the clinical user [68,69] or whether AI developers should be held solely or share accountability [68]. Developing a widely accepted consensus on this will help guide the implementation of large language models for clinical use.

Recent evidence suggests that many different types of legal and regulatory responses could be used to reduce ambiguity and support AI adoption by health care providers or systems. Importantly, at least some of these responses would not require passing new legislation or creating new regulatory frameworks at either the state or federal level, an important but always difficult lever to use. Consider the ways existing legal approaches, such as contract law, may allow developers and implementing facilities to resolve current uncertainties [70,71]. For instance, licensing agreements between health care systems and AI developers could include terms regarding liability and indemnification, and require developers to facilitate risk assessment and safety monitoring. Other private actors, such as the Coalition for Health AI (as one illustrative example), may also play a convening role in the development of best practices around the use of AI in health care settings, such as through the creation of its Responsible AI Guide and Checklist.

Our findings underscore that efforts to build health care providers’ capability and opportunity to use AI DHTs must directly address pervasive concerns about existential threat, skill erosion, and the loss of the human element in clinical care. Across reviews, providers expressed fears that AI could displace clinical roles, undermine professional identity, or lead to deskilling through overreliance on automated recommendations, particularly when AI output conflicted with clinical judgment or was poorly explained. These concerns are not simply attitudinal barriers but reflect rational responses to opaque systems, unclear accountability, and limited guidance on how responsibility is shared between clinicians and AI tools. This review identifies that building trust in AI outputs to support clinician capability may be done through improving model interpretability and providing training and knowledge on AI processes and outputs.

Recent literature shows that current implementation efforts that focus narrowly on technical training may be insufficient. Instead, strategies that explicitly frame AI as an augmentative—not substitutive—DHT are needed, emphasizing the clinician’s role in contextualizing AI output, maintaining ultimate decision-making authority, and preserving clinical expertise [72]. Educational initiatives could incorporate case-based learning that addresses disagreement between AI and clinicians, ethical decision-making, and strategies to avoid automation bias, thereby supporting skill retention and critical appraisal rather than passive reliance on AI [73]. To protect the human interaction at the core of health care, implementation strategies could also prioritize workflow designs in which AI reduces administrative burden and cognitive load, allowing providers to spend more time on relational aspects of care, such as communication, empathy, and shared decision-making [72]. Organizational policies and governance structures that clarify accountability, support reflective practice, and legitimize clinician discretion to override AI recommendations may further mitigate fears of job loss and loss of professional autonomy. Together, these strategies highlight that supporting providers’ capability and opportunity for AI use requires coordinated investments across education, organizational infrastructure, and policy environments, rather than reliance on individual-level motivation alone [74].

The integration of AI into clinical care challenges existing implementation science paradigms. Our findings demonstrate the need to adapt or extend frameworks such as CFIR to capture AI-specific constructs, including AI transparency, model lifecycle dynamics, data governance, and emergent psychological determinants, such as existential threat and relational components. Implementation strategies for AI must therefore be multidimensional, addressing technical optimization, clinician engagement, organizational readiness, and regulatory context simultaneously. Of note, several CFIR constructs, including trialability (innovation); need and motivation (implementer); relational connections, communications, and relative priority (inner setting); and external pressure (outer setting), were not identified in any of the included reviews. This absence may reflect that these determinants are not as salient for AI digital tools as for other interventions; however, this may be an artifact of how determinants were extracted and the language with which these types of determinants may be described in the literature on AI tools.

### Limitations and Strengths

This umbrella review has some important limitations. The review synthesized evidence from existing reviews rather than primary studies, which may have amplified commonly reported determinants while obscuring contextual nuance, temporal changes, or setting-specific dynamics present in individual implementation efforts. Furthermore, the quality, scope, and methodological rigor of the included reviews varied, and many reviews did not clearly specify health care settings, geographic contexts, or theoretical frameworks, limiting our ability to draw conclusions about how implementation determinants may differ across health systems, populations, or regulatory environments. For example, although we identified inner setting resources and geography as potential determinants, we cannot draw systematic conclusions about differences in determinants across settings or geographic locations. Furthermore, we were unable to extract the phase of implementation or assess the dynamic nature of determinants, which can evolve over time. We used vote counting for how many reviews described CFIR barriers and facilitators; however, this method does not account for varying size and quality of the included reviews. Five of our reviews had quality ratings of 60% or lower, which may limit the rigor of our findings. We conducted a sensitivity analysis, which showed that excluding these reviews resulted in a change of 12% to 16% count of reviews in each CFIR level but did not qualitatively change conclusions drawn as the CFIR levels and constructs continued to be represented by other reviews.

The review was limited to English-language publications and peer-reviewed reviews, potentially excluding relevant gray literature, industry reports, and non-English evidence that may capture more pragmatic or rapidly evolving implementation experiences, particularly in low- and middle-income or non-Western health system contexts. In this study, coding sought to capture what was explicitly stated in the articles and may not have captured relationships among constructs or implicit barriers or facilitators (eg, provider trust may lead to lower motivation among clinicians to use AI). Although we pilot tested the extraction protocol, used a dual-blinded review process, and held regular consensus meetings to resolve discrepancies between extractions, there may still be variation in how review results were interpreted through CFIR. On the basis of how results were presented in reviews, it was not always possible to discern if a determinant was a barrier, facilitator, or both. If it was not possible to determine whether the determinant was a barrier or a facilitator, we coded it as both. Some reviews indicated that a specific determinant was both a barrier and a facilitator. We also coded these determinants as both.

This study has several notable strengths. First, the use of an umbrella review design enabled a comprehensive and high-level synthesis of a rapidly expanding and heterogeneous body of literature on AI implementation in clinical settings, providing a consolidated evidence base that would be difficult to achieve through primary studies alone. Second, grounding the synthesis in CFIR allowed for systematic, theory-informed organization of multilevel implementation determinants, enhancing conceptual clarity and facilitating translation of findings into actionable implementation strategies. Third, the combination of deductive mapping to CFIR constructs with inductive identification of emergent, AI-specific determinants represents a key strength, as it both leverages established implementation science theory and advances it by identifying constructs—such as transparency, accountability, trust, model bias, and professional identity threat—that may not be fully captured in existing frameworks. Fourth, the review used rigorous and transparent methods, including protocol registration in PROSPERO, comprehensive database searches with an updated search window, blinded dual independent screening and data extraction, consensus procedures, and formal quality assessment using the JBI tool, which together strengthen the credibility and reproducibility of the findings.

### Conclusions

Effective and equitable AI implementation requires acknowledging and addressing a broad constellation of determinants at the innovation, implementer, inner setting, and outer setting levels. AI introduces unique technical and ethical complexities that exceed the boundaries of existing implementation frameworks. As health systems increasingly adopt AI tools, tailored implementation approaches that emphasize transparency, trust-building, ongoing model monitoring, and policy alignment will be essential to ensure safe and sustainable use in diverse clinical environments.

## Supplementary material

10.2196/101509 Multimedia Appendix 1 Search strategy.

10.2196/101509 Multimedia Appendix 2 Inclusion criteria.[Aff aff1]

10.2196/101509 Checklist 1 PRIOR checklist.

## Abbreviations

CCA: corrected covered area
CFIR: Consolidated Framework for Implementation Research
DHT: digital health tool
FDA: Food and Drug Administration
GROOVE: Graphical Representation of Overlap for Overviews
JBI: Joanna Briggs Institute
ML: machine learning
PRIOR: Preferred Reporting Items for Overview of Reviews
PRISMA: Preferred Reporting Items for Systematic Reviews and Meta-Analyses
TMF: theory, model, or framework
