# Supplementary material for: AI-Enabled Digital Health Tool Implementation in Clinical Practice Settings: Umbrella Review
Source: JMIR AI. 2026 Nov 4;5:e101509. doi: 10.2196/101509 (PMC13734705; doi:10.2196/101509)
Supplement: Supplementary file 2 [file ai-v5-e101509-s001.docx]

**PUBMED**

Filters: Systematic Review; Publication Date – 2013-2025

"Computers, Handheld"[Mesh] OR "User-Computer Interface"[Mesh] OR "Telephone"[Mesh:NoExp] OR "Cell Phone"[Mesh] OR "Artificial Intelligence"[Mesh:NoExp] OR "Machine Learning"[Mesh] OR "Telemedicine"[Mesh] OR "biomedical technology"[Mesh] OR "medical informatics"[Mesh] OR "remote sensing technology"[Mesh:NoExp] OR "electronic health records"[Mesh] OR "wearable electronic devices"[Mesh] OR "mobile applications"[Mesh] OR "handheld computer*"[tiab] OR "User-Computer Interfac*"[tiab] OR "telephone*"[tiab] OR “cell phone*”[tiab] OR "Artificial Intelligence"[tiab] OR "Machine Learning"[tiab] OR "Telemedicine"[tiab] OR "biomedical technolog*"[tiab] OR "medical informatic*"[tiab] OR "remote sensing technolog*"[tiab] OR "electronic health record*"[tiab] OR "wearable electronic device*"[tiab] OR "mobile applicat*"[tiab] OR "artificial intelligence"[tiab] OR "augmented reality"[tiab] OR "cellular phone*"[tiab] OR "clinical decision support"[tiab] "computerised medical record*"[tiab] OR "computerized medical record*"[tiab] OR "decision support system*"[tiab] OR "deep learning"[tiab] OR "digital health*"[tiab] OR "digital therapeutic*"[tiab] OR ehealth[tiab] OR "e health"[tiab] OR "electronic health record*"[tiab] OR "electronic medical record*"[tiab] OR EMR[tiab] OR "health application*"[tiab] OR "health app*"[tiab] OR "health informatic*"[tiab] OR "health information technology"[tiab] OR "health IT"[tiab] OR "health tech*"[tiab] OR "health wearable*"[tiab] OR "health care application*"[tiab] OR "health care apps"[tiab] OR "health care informatic*"[tiab] OR "health care information technology"[tiab] OR "health care IT"[tiab] OR "health care technolog*"[tiab] OR "healthcare apps"[tiab] OR "healthcare informatic*"[tiab] OR "healthcare information technology"[tiab] OR "healthcare IT"[tiab] OR "healthcare technolog*"[tiab] OR "healthcare wearable*"[tiab] OR "healthcare IoT"[tiab] OR healthtech[tiab] OR "internet of things"[tiab] OR "machine intelligence"[tiab] OR "machine learning"[tiab] OR "medical informatics"[tiab] OR mhealth[tiab] OR "m health"[tiab] OR "mobile application*"[tiab] OR "mobile app*"[tiab] OR "mobile device*"[tiab] OR "mobile health*"[tiab] OR "mobile phone*"[tiab] OR "mobile technolog*"[tiab] OR "remote monitoring"[tiab] OR "remote patient monitoring"[tiab] OR "smart phone*"[tiab] OR smartphone*[tiab] OR SMS[tiab] OR telecare[tiab] OR "tele care"[tiab] OR teleconsultation*[tiab] OR "tele consultation*"[tiab] OR telehealth[tiab] OR "tele health"[tiab] OR telemedicine[tiab] OR "tele medicine"[tiab] OR "text messag*"[tiab] OR "virtual care"[tiab] OR "virtual consultation*"[tiab] OR "virtual health care"[tiab] OR "virtual healthcare"[tiab] OR "virtual medicine"[tiab] OR "virtual systems"[tiab] OR "wearable device*"[tiab] OR "wearable electronic device*"[tiab] OR "wearable technolog*"[tiab] OR “health databases”[tiab] OR “health information technology”[tiab] OR “digital imaging”[tiab] OR “wearable sensor*”[tiab] OR “technology, medical”[tiab] OR “patient record system*”[tiab] OR “medical record*”[tiab] OR “health information management service”[tiab] OR “health information management”[tiab] OR “digital technology”[tiab] OR “health information network*”[tiab] OR "telephone triage nurs*"[tiab] AND "Diffusion of Innovation"[Mesh] OR "Diffusion of Innovation"[tiab] OR adopt*[tiab] OR advance*[tiab] barrier*[tiab] OR catalyst*[tiab] OR challenge*[tiab] OR constraint*[tiab] OR contributor*[tiab] OR deploy*[tiab] OR enabler*[tiab] OR enhance*[tiab] OR execut*[tiab] OR facilitat*[tiab] OR helper*[tiab] OR hindrance*[tiab] OR hurdle*[tiab] OR impediment*[tiab] OR implement*[tiab] OR integrat*[tiab] OR limitation*[tiab] OR pitfall*[tiab] OR promot*[tiab] OR restriction*[tiab] OR setback*[tiab] OR supporter*[tiab] OR "utilis*"[tiab] OR "utiliz*"[tiab] AND"Ambulatory Care Facilities"[tiab] OR "hospital outpatient clinic"[tiab] OR "Safety net Provider*"[tiab] OR "Academic Medical Center*"[tiab] OR "Community Health Worker*"[tiab] OR "Nurse Clinician*"[tiab] OR physician*[tiab] OR "Patient Care Team"[tiab] OR "Physician Assistant*"[tiab] OR nurse*[tiab] OR nutritionist*[tiab] OR "ambulatory care facilit*"[tiab] OR "hospital outpatient clinic*"[tiab] OR "outpatient clinic*"[tiab] OR "safety net provider*"[tiab] OR "academic medical cent*"[tiab] OR "community health worker*"[tiab] OR "nurse clinician*"[tiab] OR physician*[tiab] OR "patient care team"[tiab] OR "physician assistant*"[tiab] OR nurse*[tiab] OR nutritionist*[tiab] OR "care delivery setting*"[tiab] OR "care facilit*"[tiab] OR "child guidance clinic*"[tiab] OR "clinical care setting*"[tiab] OR "clinical environment*"[tiab] OR "clinical facilit*"[tiab] OR "clinical practice setting*"[tiab] OR "community health cent*"[tiab] OR "community mental health cent*"[tiab] OR "free standing clinic*"[tiab] OR "health care environment*"[tiab] OR "healthcare environment*"[tiab] OR "health care facilit*"[tiab] OR "healthcare facilit*"[tiab] OR "health care institution*"[tiab] OR "healthcare institution*"[tiab] OR "health care organisation*"[tiab] OR "healthcare organisation*"[tiab] OR "health care organization*"[tiab] OR "healthcare organization*"[tiab] OR "health care system*"[tiab] OR "healthcare system*"[tiab] OR hospital*[tiab] OR "hospital setting*"[tiab] OR "maternal child health cent*"[tiab] OR "medical clinic*"[tiab] OR "medical institution*"[tiab] OR "out patient clinic*"[tiab] OR "outpatient clinic*"[tiab] OR "pain clinic*"[tiab] OR "safety net clinic*"[tiab] OR "safety net hospital*"[tiab] OR surgicent*[tiab] OR "surgery cent*"[tiab] OR "teaching hospital*"[tiab] OR "treatment cent*"[tiab] OR "university hospital*"[tiab] OR "university medical cent*"[tiab] OR "urgent care cent*"[tiab] OR "urgent care clinic*"[tiab] OR "care delivery setting*"[tiab] OR "care facilit*"[tiab] OR "child guidance clinic*"[tiab] OR "mobile health units"[tiab]

**SCOPUS**

Filters: Range 2013 – 2025; Search within Article title, Abstract, Keywords

"handheld computer*" OR "User-Computer Interfac*" OR "telephone*" OR “cell phone*” OR "Artificial Intelligence" OR "Machine Learning" OR "Telemedicine" OR "biomedical technolog*" OR "medical informatic*" OR "remote sensing technolog*" OR "electronic health record*" OR "wearable electronic device*" OR "mobile applicat*" OR "artificial intelligence" OR "augmented reality" OR "cellular phone*" OR "clinical decision support" "computerised medical record*" OR "computerized medical record*" OR "decision support system*" OR "deep learning" OR "digital health*" OR "digital therapeutic*" OR ehealth OR "e health" OR "electronic health record*" OR "electronic medical record*" OR EMR OR "health application*" OR "health app*" OR "health informatic*" OR "health information technology" OR "health IT" OR "health tech*" OR "health wearable*" OR "health care application*" OR "health care apps" OR "health care informatic*" OR "health care information technology" OR "health care IT" OR "health care technolog*" OR "healthcare apps" OR "healthcare informatic*" OR "healthcare information technology" OR "healthcare IT" OR "healthcare technolog*" OR "healthcare wearable*" OR "healthcare IoT" OR healthtech OR "internet of things" OR "machine intelligence" OR "machine learning" OR "medical informatics" OR mhealth OR "m health" OR "mobile application*" OR "mobile app*" OR "mobile device*" OR "mobile health*" OR "mobile phone*" OR "mobile technolog*" OR "remote monitoring" OR "remote patient monitoring" OR "smart phone*" OR smartphone* OR SMS OR telecare OR "tele care" OR teleconsultation* OR "tele consultation*" OR telehealth OR "tele health" OR telemedicine OR "tele medicine" OR "text messag*" OR "virtual care" OR "virtual consultation*" OR "virtual health care" OR "virtual healthcare" OR "virtual medicine" OR "virtual systems" OR "wearable device*" OR "wearable electronic device*" OR "wearable technolog*" OR “health databases” OR “health information technology” OR “digital imaging” OR “wearable sensor*” OR “technology, medical” OR “patient record system*” OR “medical record*” OR “health information management service” OR “health information management” OR “digital technology” OR “health information network*” OR "telephone triage nurs*" AND “Diffusion of Innovation" OR adopt* OR advance* barrier* OR catalyst* OR challenge* OR constraint* OR contributor* OR deploy* OR enabler* OR enhance* OR execut* OR facilitat* OR helper* OR hindrance* OR hurdle* OR impediment* OR implement* OR integrat* OR limitation* OR pitfall* OR promot* OR restriction* OR setback* OR supporter* OR "utilis*" OR "utiliz*" AND "Ambulatory Care Facilities" OR "hospital outpatient clinic" OR "Safety net Provider*" OR "Academic Medical Center*" OR "Community Health Worker*" OR "Nurse Clinician*" OR physician* OR "Patient Care Team" OR "Physician Assistant*" OR nurse* OR nutritionist* OR "ambulatory care facilit*" OR "hospital outpatient clinic*" OR "outpatient clinic*" OR "safety net provider*" OR "academic medical cent*" OR "community health worker*" OR "nurse clinician*" OR physician* OR "patient care team" OR "physician assistant*" OR nurse* OR nutritionist* OR "care delivery setting*" OR "care facilit*" OR "child guidance clinic*" OR "clinical care setting*" OR "clinical environment*" OR "clinical facilit*" OR "clinical practice setting*" OR "community health cent*" OR "community mental health cent*" OR "free standing clinic*" OR "health care environment*" OR "healthcare environment*" OR "health care facilit*" OR "healthcare facilit*" OR "health care institution*" OR "healthcare institution*" OR "health care organisation*" OR "healthcare organisation*" OR "health care organization*" OR "healthcare organization*" OR "health care system*" OR "healthcare system*" OR hospital* OR "hospital setting*" OR "maternal child health cent*" OR "medical clinic*" OR "medical institution*" OR "out patient clinic*" OR "outpatient clinic*" OR "pain clinic*" OR "safety net clinic*" OR "safety net hospital*" OR surgicent* OR "surgery cent*" OR "teaching hospital*" OR "treatment cent*" OR "university hospital*" OR "university medical cent*" OR "urgent care cent*" OR "urgent care clinic*" OR "care delivery setting*" OR "care facilit*" OR "child guidance clinic*" OR "mobile health units" AND Systematic AND Review

**CINAHL**

FIlters: Published Date January 2013 – June 2025, Publication Type Systematic Review

(MH "Mobile Health Units") OR (MH "Telehealth") OR (MH "Cellular Phone") OR (MH "Mobile Applications") OR (MH "Health Information Networks") OR (MH "Health Information Systems") OR (MH "Databases, Health") OR (MH "Electronic Health Records") OR (MH "Patient Record Systems") OR (MH "Medical Records") OR (MH "Health Information Management Service") OR (MH "Health Information Management") OR (MH "Technology, Medical") OR (MH "Medical Informatics") OR (MH "Wearable Sensors") OR (MH "Artificial Intelligence") OR (MH "Digital Health") OR (MH "Digital Technology") OR (MH "Digital Imaging") OR (MH "Computers, Hand-Held") OR (MH "Telephone Triage Nursing") AND MH (MH "Diffusion of Innovation") OR TI ( "Diffusion of Innovation" OR adopt* OR advance* barrier* OR catalyst* OR challenge* OR constraint* OR contributor* OR deploy* OR enabler* OR enhance* OR execut* OR facilitat* OR helper* OR hindrance* OR hurdle* OR impediment* OR implement* OR integrat* OR limitation* OR pitfall* OR promot* OR restriction* OR setback* OR supporter* OR "utilis*" OR "utiliz*" ) OR AB ( "Diffusion of Innovation" OR adopt* OR advance* barrier* OR catalyst* OR challenge* OR constraint* OR contributor* OR deploy* OR enabler* OR enhance* OR execut* OR facilitat* OR helper* OR hindrance* OR hurdle* OR impediment* OR implement* OR integrat* OR limitation* OR pitfall* OR promot* OR restriction* OR setback* OR supporter* OR "utilis*" OR "utiliz*" ) AND MH ( (MH "Ambulatory Care Facilities") OR (MH "Surgicenters") OR (MH "Ambulatory Care") ) OR TI ( "Ambulatory Care Facilities" OR "hospital outpatient clinic" OR "Safety net Provider*" OR "Academic Medical Center *" OR "Community Health Worker*” OR "Nurse Clinician*” OR "Physicians " OR "Patient Care Team" OR "Physician Assistant*” OR "Nurse*” OR "Nutritionist*” OR "ambulatory care facilit*" OR "hospital outpatient clinic*" OR "outpatient clinic*" OR "safety net provider*" OR "academic medical cent*" OR "community health worker*" OR "nurse clinician*" OR "physician*" OR "patient care team" OR "physician assistant*" OR "nurse*" OR "nutritionist*" OR "care delivery setting*" OR "care facilit*" OR "child guidance clinic*" OR "clinical care setting*" OR "clinical environment*" OR "clinical facilit*" OR "clinical practice setting*" OR "community health cent*" OR "community mental health cent*" OR "free standing clinic*" OR "health care environment*" OR "healthcare environment*" OR "health care facilit*" OR "healthcare facilit*" OR "health care institution*" OR "healthcare institution*" OR "health care organisation*" "healthcare organisation*" OR "health care organization*" OR "healthcare organization*" OR "health care system*" OR "healthcare system*" OR hospital* OR "hospital setting*" OR "maternal child health cent*" OR "medical clinic*" OR "medical institution*" OR "out patient clinic*" OR "outpatient clinic*" OR "pain clinic*" OR “safety net clinic*” OR “safety net hospital*” OR surgicent* OR “surgery cent*” OR "teaching hospital*" OR "treatment cent*" OR "university hospital*" OR "university medical cent*" OR "urgent care cent*" OR "urgent care clinic*" OR "care delivery setting*" OR "care facilit*" OR "child guidance clinic*" ) OR AB ( "Ambulatory Care Facilities" OR "hospital outpatient clinic" OR "Safety net Provider*" OR "Academic Medical Center *" OR "Community Health Worker*” OR "Nurse Clinician*” OR "Physicians " OR "Patient Care Team" OR "Physician Assistant*” OR "Nurse*” OR "Nutritionist*” OR "ambulatory care facilit*" OR "hospital outpatient clinic*" OR "outpatient clinic*" OR "safety net provider*" OR "academic medical cent*" OR "community health worker*" OR "nurse clinician*" OR "physician*" OR "patient care team" OR "physician assistant*" OR "nurse*" OR "nutritionist*" OR "care delivery setting*" OR "care facilit*" OR "child guidance clinic*" OR "clinical care setting*" OR "clinical environment*" OR "clinical facilit*" OR "clinical practice setting*" OR "community health cent*" OR "community mental health cent*" OR "free standing clinic*" OR "health care environment*" OR "healthcare environment*" OR "health care facilit*" OR "healthcare facilit*" OR "health care institution*" OR "healthcare institution*" OR "health care organisation*" "healthcare organisation*" OR "health care organization*" OR "healthcare organization*" OR "health care system*" OR "healthcare system*" OR hospital* OR "hospital setting*" OR "maternal child health cent*" OR "medical clinic*" OR "medical institution*" OR "out patient clinic*" OR "outpatient clinic*" OR "pain clinic*" OR “safety net clinic*” OR “safety net hospital*” OR surgicent* OR “surgery cent*” OR "teaching hospital*" OR "treatment cent*" OR "university hospital*" OR "university medical cent*" OR "urgent care cent*" OR "urgent care clinic*" OR "care delivery setting*" OR "care facilit*" OR "child guidance clinic*" )
