# Supplementary material for: AI-Enabled Digital Health Tool Implementation in Clinical Practice Settings: Umbrella Review
Source: JMIR AI. 2026 Nov 4;5:e101509. doi: 10.2196/101509 (PMC13734705; doi:10.2196/101509)
Supplement: Supplementary file 3 [file ai-v5-e101509-s002.docx]

Supplement 1: Inclusion/Exclusion Criteria

|  | **Include** | **Exclude** |
| --- | --- | --- |
| **Year Published** | 2013 to 2025 | Before 2013 |
| **Language** | English | Non-English |
| **Country** | All | No exclusions |
| **Study Type** | Systematic Review/Scoping Review/Literature Review/Narrative Review/ Rapid Review/ Meta-analysis/Literature Analysis | All other study types: observational studies (cohort, case-control, cross-sectional, case-crossover, ecologic, case series, case reports); qualitative studies; commentary or debate papers; conference abstracts; protocol papers (with no reported outcomes) |
| **Intervention Targets** | Clinical care settings e.g., ambulatory care facilities, hospital outpatient clinic, Safety net Provider, academic medical centers, etc. | Non-clinical care settings e.g., home, school, long-term care facilities, imaging and radiology centers, rehab centers, mental health facilities. |
| **Intervention Type** | Digital health tool (DHT) including electronic health records (EHRs), AI, computerized decision support systems, telemedicine, smartphone applications, websites, wearable devices, text messaging, health monitoring systems, and telephone support. | Does not include HIT or includes technologies that are outside our scope, e.g., blockchain technologies |
| **Area of Focus** | Primarily addresses barriers (challenges, hinderances, etc.) and facilitators (advantages, enablers, etc.)  to implementing digital health tools. | Does not address barriers and facilitators to implementing digital health OR presents them in passing (e.g., not one of the review aims). |
| **Intended users/adopters of the digital health tool** | Healthcare providers (doctors, nurses, nurse practitioners, social workers, dieticians, physical therapists, exercise physiologists, medical assistants, clinical research assistants, community health workers etc.) | Non-healthcare providers (patients, caregivers, medical students, administrators). |
